# The Interplay of EFL Students’ Enjoyment, Hope, Pride and Self-Regulation

**DOI:** 10.3389/fpsyg.2021.803476

**Published:** 2021-12-06

**Authors:** Xuehong Yin

**Affiliations:** School of Accounting, Zhejiang Gongshang University, Hangzhou, China

**Keywords:** EFL students’ enjoyment, hope, pride, self-regulation, EFL

## Abstract

Nowadays, emotions are among the most significant issues in the route of learning a language that should be taken into consideration. Consistent with the fundamental function of positive psychology (PP) and also the theory of broaden-and-build, enjoyment in language learning especially the foreign language is among those positive emotions that encourage EFL learners to develop their perspective to achieve. Efforts to apprehend and develop the academic achievement of EFL learners have also progressively concentrated on self-regulation as it boosts learners’ enjoyment, hope, pride, self-control, and learning. Besides, in line with the investigations of these positive emotions, principles of PP, the present review makes every effort for the interplay and its effects in language learning. It is pertinent to state that the present review of studies can benefit academic organizations, professional development centers, policymakers in the academic community to consider the role of emotions, namely positive ones and their positive impact on language learning.

## Introduction

The process of learning is accompanied by feelings such as enjoyment, hope, excitement, fear, and fatigue, and those learners who experience such feelings are more likely to learn, perform well, and develop their sense of identity (Pekrun and Linnenbrink-Garcia, 2014; MacIntyre, 2016). Researchers have mainly focused their research on unpleasant and aversive feelings, particularly language apprehension, boredom, and anxiety (Dewaele and Macintyre, 2014; Daubney et al., 2017; Kruk and Zawodniak, 2020; Pawlak et al., 2020; Derakhshan et al., 2021). In the early years of study, feelings were deemed as secondary variables, and only some feelings, such as fear, were examined. The perception of feelings has changed since the 2000s. They are now considered an active force that promotes learning and guides decision-making (Park et al., 2014). Dornie and Ryan (2015) asserted that feelings have generally been disregarded by foreign language learning experts, regardless of their importance to our lives. They added that this is due to the psychological history in the field and that foreign language learning research needs to address this issue. In the study of foreign languages, feelings are essential to the educational process, since without them learning would not be adequate (Dewaele, 2015). Psychologists began turning scholars’ attention to people’s feelings of well-being with the advent of behavioral psychology (Seligman and Csikszentmihalyi, 2000). The function that feelings have in the lives of individuals, as well as their close connection with thinking, makes their influence on learning somewhat surprising. The new awareness prompted by the rise of positive psychology has stimulated efforts to understand these effects on learning (MacIntyre, 2016; MacIntyre et al., 2019; Wang et al., 2021), and positive feelings can greatly improve learning, as evidenced in recent research, so denying their importance in language acquisition is no longer possible (Seligman and Csikszentmihalyi, 2000).

Due to the key role that emotion plays in the process of learning a language, many investigations (e.g., Dewaele and Alfawzan, 2018; Dewaele et al., 2019), have examined the role positive feelings might play in educational success and second language learning. In educational environments, learners encounter a wide range of feelings that impact their performance and outlook. Results from studies by Pekrun et al. (2002) demonstrated that behavioral and psychological studies in academia can enhance a range of emotions in education environments by referring to different feelings felt by learners in classrooms. These studies on education are significant for enhancing learners’ engagement, teaching methods, intellectual capabilities, autonomy, and educational success. In addition to affecting the social climate in courses and academic settings, feelings can affect learners’ performance, enthusiasm, motivation, and professional advancement (Pekrun, 2006). According to Pekrun et al. (2007), as a result of achievement outcomes, educational feelings can be defined either as stress, satisfaction, guilt, etc. or as feelings connected to achievement and disappointment. Learning processes are influenced by some other emotions such as expectations, fatigue, rage, shame, and hope, other than those related to achievement and task demands. There are also other emotions present in academic environments, such as happiness, self-esteem, frustration, optimism, and fatigue, in addition to enjoyment (Pekrun and Linnenbrink-Garcia, 2014; Wang et al., 2021).

Indeed, regarding the role of positive feelings in second language learning, enjoyment has frequently been mentioned as one of the feelings that are used often to explain the enjoyableness of a hobby, career, school, or project (Li et al., 2018; Jiang and Dewaele, 2019). When one accomplishes an activity or acquires something, one feels the fulfillment and pleasure that comes with it (Dewaele and Macintyre, 2014). People defined enjoyment as a feeling that they feel when they do anything that challenges them further than what they are currently capable of Seligman and Csikszentmihalyi (2000). Second language enjoyment is described by Boudreau et al. (2018) as an internal feeling that is independent of external satisfaction.

In addition to the rise of self-esteem in effective learning, which is described as the sense of dignity and individual worth, the role pride plays in effective learning is at the center of attention (Walden, 2009). When one realizes they have achieved positive results independently, the emotion that arises is joy and pride (Tiedens et al., 2000). According to Pekrun’s control-value theory, happiness is an encouraging emotion that enhances your probability of success. The researchers found that pride is linked with a physical response that is commonly perceived and expressed automatically during pride experiences (Tracy and Robins, 2007). In positive learning, it is obvious that hope also plays an increasingly significant role (Lopez et al., 2009). Aspirations are all kinds of goals that people want to achieve under the term hope. According to this theory, people with high degrees of hope are more likely than those with low degrees of hope to find effective strategies to overcome a demanding situation and help them achieve their goals (Lopez et al., 2009). Conversely, high-hope people are much more possible to exhibit positive feelings than low-hope people (Snyder, 2002). Educational success has also been linked to hope (Snyder et al., 2000). People’s hope levels vary, depending on their experiences (Webb, 2013). Those with more hope are more likely to be educationally prosperous, which in turn produces a healthy feeling of pride and enthusiasm, whereas those with less hope are less likely to achieve, resulting in loss of confidence (Horton and Wallander, 2001; Lopez, 2013). Pekrun (2006) control-value theory of feelings has been gaining significant interest within the realm of academic studies and based on this theory, the influences of the emotions of achievement on education and performance rely on the interaction of numerous mediating processes, like learners’ inspiration, use of strategies, and control of education (Pekrun, 2006). Feelings are believed to affect learners’ intrinsic inspiration to learn, based on awareness and interest to learn, along with their extrinsic inspiration to succeed or avoid failure (Pekrun et al., 2011). Specifically, activating constructive emotions like joy, aspiration, and pride is assumed to promote not only intrinsic but also extrinsic inspiration and maintain self-regulation, which has a positive influence on educational presentation. A key element of self-regulation is self-awareness as educational achievement depends on learners managing their thoughts, actions, and feelings during class time. In addition to controlling behavior, thinking, and feelings, self-regulation impacts our actions (Duru et al., 2014). From a socio-cognitive viewpoint, investigation on self-regulation has emphasized a person’s ability to oversee and alter practice, cognition, impact, and situation to attain an objective (Efklides et al., 2002).

Learning second languages is easier for learners who are motivated by good feelings, as they can notice the things in the educational setting and are more mindful of language input (Dewaele and Alfawzan, 2018). They argued that pleasant feelings can reduce negative emotions which is important because negative feelings impair concentration and limit verbal input. As Pekrun and Linnenbrink-Garcia (2014) stated, as educators and learners spend so many hours together in such an emotional setting, and they develop relationships that enable them to relate to each other on many levels to experience many feelings together. As a result, academic environments may be described as places where feelings such as enjoyment, hope, self-esteem, frustration, guilt, fatigue, and stress exist. Biologically and mentally, the feeling is generally categorized as a state of mind which is sudden, abrupt, strong, and temporary, and usually relates to a specific event or circumstance (Colman, 2009). As MacIntyre and Gregersen (2012) affirmed, good feelings reduce the adverse effects of unpleasant feelings and enhance the capacity for language input, while unpleasant feelings adversely affect the ability to pay attention. According to Fredrickson (2003), broaden and build theory, for example, good feelings all can broaden individuals’ immediate decision-making capabilities and support the development of their long-term human potential, including physiological, cognitive, and emotional capacities.

The control-value theory proposed that the influences of reaching emotions on education and accomplishment count on the relationship of numerous facilitating process, like learners’ enthusiasm, strategy practice, and guideline of education (Pekrun, 2006). Emotions seem to affect learners’ inherent enthusiasm to acquire that is in line with interest and curiosity in learning, along with their extrinsic motivation associated with the self-actualization of accomplishment or the prevention of disaster (Pekrun et al., 2011). Precisely, constructive stimulating feelings such as enjoyment, hope, and pride are supposed to arouse not only intrinsic but also extrinsic inspiration and maintain self-regulation, as a result confidently influencing theoretical presentation (Wang and Guan, 2020).

Based on the above-mentioned points, although the role of unfavorable feelings in second language instruction and learning has received considerable attention, positive emotions have been largely ignored (Dewaele and Macintyre, 2014) and there is still significant ambiguity regarding the connection between second/foreign language learning and good feelings emotions (Matsuda and Gobel, 2004; Liu, 2006; Wang and Guan, 2020). Founded on the aforementioned points and their noteworthy consequence on learning, the present review makes efforts to take some of these positive emotions such as hope, pride enjoyment, and self-regulation into account and scrutinize how they can work as mediating functions in the process of foreign/second language learning. Indeed, based on the researcher’s knowledge, there was no review of literature that has investigated enjoyment, pride and hope, and self-regulation all together in language education.

## Review of Literature

### Positive Psychology in Education

Due to improvements in their standard of living, people’s demands for education have changed substantially in recent years. Education systems are expected to prepare intellectually capable learners and to take care of their emotional well-being in the 21st century (Whiteside et al., 2017). The positive behavior movement has a strong impact on academic achievement due to recent research about 21stcentury skills regarding its effect on scholastic research. Positive teaching is the implementation of positive psychology in the academic setting (Pluskota, 2014). Several definitions of positive learning have been proposed such as Seligman et al. (2009) definition, which refers to education that stresses both academic success and learners’ happiness. They defined effective learning as teaching basic skills and teaching enjoyment. In other words, successful learning refers to academic solutions that place equal weight on academic achievement and personal growth. Their research shows that effective learning leads to better academic performance by cultivating favorable feelings in learners. Another definition of emotion given by Keltner et al. (2013) is multiple reactions to situations that are perceived as positive or negative in our inner or outer surroundings, situations that matter to our purpose. Language learning is facilitated by pleasant feelings, so educators should boost learners’ enjoyment rather than focus on decreasing stress. Giving psychological support in language classes is a way that educators can help learners handle their feelings. When educators identify especially the sources of unpleasant feelings, they can use self-regulation methods such as self-control to overcome them. When educators recognize particularly the origins of unfavorable feelings, they can create an emotionally supportive environment. Learners may also benefit from providing psychological support during their educational process. The accomplishment was positively correlated with both enjoyment and self-esteem. The self-control component was positively associated with academic success among learners reporting greater amounts of both positive feelings. The results indicate that, for learners who report a lack of pride and enjoyment, their judgment does not affect achievement; and for learners who report a lack of satisfaction, it has a poor relationship with success (Villavicencio and Bernardo, 2013).

### Enjoyment

The feeling of enjoyment contrasts with sadness, and it is one of the essential human feelings (Plutchik, 2001). Pekrun’s control-value theory defines it as a favorable positive feeling that illustrates physical activity, and one that is associated with accomplishment. Dewaele and Macintyre (2014) defined enjoyment as a powerful feeling that motivates the human drive for success despite obstacles and insufficient skills. It is generally experienced by people when they are striving for and investing in an outcome that is meaningful to them. Whenever a person goes beyond themselves and achieves something unexpected, they can feel a sense of excitement or enjoyment (Csikszentmihalyi, 2008). A flow state is defined by deep concentration and commitment in a particular activity without conscious awareness of time or self, and it is essential for second language education and acquisition (Li et al., 2018). Moreover, enjoyment in a foreign language, namely called (FLE), is described as being psychologically favorable and motivating, causing learners to become engaged and enthusiastic (Dewaele and Macintyre, 2014). A situation in which learners not only meet their desires but also surpass them to undertake something unpredicted in the process of learning a second language (Dewaele and Macintyre, 2014). Having fun can positively affect the long-term resilience and resiliency of learners because it is such an essential part of a positive experience. An individual is presumed to gratify the route of foreign language learning when he or she is responding with joy to personal situations and responsibilities (Goetz et al., 2006). Foreign language enjoyment is greatly influenced by its scholastic cycle, like associating with caring and cooperative peers, along with interacting with eager language teachers who offer a diversity of interesting and demanding class activities for the sake of involving students (Pavelescu and Petric, 2018).

### Hope

The concept of hope can be described by Snyder (2002) as the combination of the perception of abilities to achieve desired goals and the perception of desire to use those abilities. Snyder’s theory explains how individuals achieve their goals by defining them, establishing a plan to reach them (pathways), and determining their purpose (agency) (Lopez et al., 2009). Likewise, hopeful learners have higher achievement aspirations, as well as more optimistic outlooks about the future (Snyder et al., 2006). The relationship between hope and depression is reversed, as is the relationship between hope and behavioral problems and signs of pain or school alienation (Gilman et al., 2006). In addition, hope is often associated with educational success, with the premise that students who are highly motivated to work toward their objectives will exert more effort and perseverance. Further, a positive outlook affects purpose-driven actions indirectly through personal responsibility because high hopes learners are better at handling pressure, controlling emotional reactions to follow appropriate ones toward success, recognizing and handling obstacles, and refocusing efforts to achieve outcomes with greater effectiveness; they are more capable of solving problems (Snyder, 2002).

### Pride

Concerning Bandura’s theory, Zimmerman and Cleary (2009) suggest that self-regulatory criticism loops depend on three closely related iterative cycles: introspection, discernment, and self-reaction. Based on this standpoint, every one of these cycles is affected by self-reactions to individual criticism. Pride, which is a positive individual emotion that can aid ongoing learning efforts, could be an example of self-reaction. Zimmerman and Cleary (2009) do not explicitly mention pride but they proved that a student’s awareness of gratification and fulfillment with their presentation and the parallel positive impact encourages them to go on with their learning efforts. In addition, learners who are very complacent are more ready to make adaptive interpretations about mistakes and to conclude that the next time they face negative consequences, they must resort to strategies that differ to be more successful. More self-satisfied students ultimately exhibit higher self-efficacy and greater philosophies about the value of the task, leading to more self-regulated learning practices in the task cycle’s future iterations. While this research does not directly estimate pride, it proposed that positive self-assessment is associated with better self-regulation, a discovery that is close to pride construct. Furthermore, Pekrun et al. (2002) demonstrated that pride is associated with a more extensive utilization of metacognitive techniques, organization, expansion, and critical thinking and it can be associated with greater self-regulation and higher performance.

### Self-Regulation

Adjusting mental processes, attitudes, and behaviors in a systematic, sequential manner to achieve individual goals is referred to as self-regulation (Zimmerman, 2002). It is generally defined by Zimmerman (2002) as the amount of intellectual, emotional, and psychological engagement in education and accomplishment of goals by learners. Indeed, self-regulation alludes to self-generated ideas, emotions, and activities that are systematically prearranged and adjusted as required to influence an individual’s education and inspiration, and enthusiasm (Schunk and Ertmer, 2000). Learners’ self-regulation can be defined as the process by which they consistently direct their thoughts, actions, and outcomes toward achieving their purpose (Schunk et al., 2008). A learner’s self-generated ideas and actions that aim to accomplish academic goals and require the learner to take action as part of the educational setting are considered self-regulation that deals with more than the acquisition of skills, as it involves a consciousness of one’s growth that allows one to adjust performance and attitudes to be a better student and to acquire more professionally (Colthorpe et al., 2019). Self-regulated learning, as defined by Pintrich (2000), is an engaged, practical approach that enables learners to set their learning objectives and monitor, manage and adjust their behavior, enthusiasm, and thinking as they achieve them. A study has shown that academic success is strongly influenced by self-regulation (Kızıl and Savran, 2018; Shih et al., 2019). It is stated that even language skills such as writing skills can be improved by using self-regulation techniques (Graham and Harris, 2000). In addition, proficient readers are known to use methods like deductions and summaries as a way to regulate their learning strategy (Butler, 2002; Wang and Guan, 2020). Organizing, observing, and analyzing mental performance behaviors are frequently employed in handling difficulties (Araghian et al., 2018). When motivated to reach a target, learners engage in self-regulation as a way of helping them reach their goals. Motivation comes from a feeling of accomplishment resulting from self-regulation, and this keeps learners engaged (Pintrich, 2000; Zimmerman and Schunk, 2011). Learners who are diligent in and conscious of their educational growth are known as self-regulatory learners. They are more expected to persist and attain better outcomes when confronted with a challenging scholastic environment (Zusho, 2017).

## Implications and Future Directions

The appearance of PP in second language acquisition (SLA) and the explicit area of emotion and language learning echoes with an alteration in mindset among L2 scholars, moving from negative emotions to a more positive and well-adjusted method for the study of language instruction and learning. Indeed, PP is considered an interesting issue to explore the place of emotions in SLA, as it includes dimensions such as constructive associations, traits, and emotions, which are fundamental to exploring and nurturing positive affective involvements in language teaching which are of crucial applicability to the examination and cultivation of valuable emotional practices in language teaching space. A preliminary survey in this potential arena already provides new thoughts, information, and perceptions for theorists and suggests practical interventions for language educators (Dewaele et al., 2019; MacIntyre et al., 2019). High positive feelings are characterized by enjoyment and enthusiasm, and high positive feelings also lead to a pleasant mood and more optimism during activities. The result is a more positive outlook. Constructive feelings enhance learners’ attention, self-regulated education, boredom levels, and propensity for life-long learning (Oates, 2019). The use of flexible learning methods, autonomy, and cognitive capabilities for task engagement is likely to be positively influenced by pleasant feelings such as enjoyment, optimism, and self-esteem (Pekrun et al., 2011). The enthusiasm of students can be influenced in the present and the long term by how much they take pleasure in learning or enjoy (Chatzistamatiou et al., 2015). Since emotions can trigger various forms of information processing and problem-solving and enhance or hinder learners’ self-regulation in learning, it can be said that they affect the more cognitive dimensions of education (Pekrun et al., 2002).

A major outcome in every academic setting is educational success, and positive feelings have been linked strongly to educational performance according to studies conducted in this field. This indicates that educators should pay attention to the feelings of learners. For English educators, the current review of the literature suggests that they should take learners’ emotional reactions toward learning English into consideration since if they do not, they will not be able to minimize the unpleasant feelings and increase the positive ones. In addition, educators must understand that the utilization of certain language abilities brings out varied feelings, even in the same student. Furthermore, teachers should take into account the class reactions to learning English and assist them, when necessary, as well as try to eliminate unpleasant feelings such as frustration, fear, discouragement, and fatigue. When feelings are integrated into learning, both teachers and learners can enjoy more, experience less stress, anger, and disappointment. Encouraging enjoyment, especially in subjects that students like and feel good about in the class, could also enhance the language educational experience in the scholastic environment. Furthermore, students with high enjoyment of English as a foreign language had a high level of performance and self-regulation than students with medium to low enjoyment. Educators’ attitudes and positive classroom environment were common sources of enjoyment mentioned by learners. Therefore, teachers must reduce negative feelings and encourage positive ones in the classroom for the sake of optimizing the language educational experience and they must develop a positive atmosphere in their class by utilizing non-aggressive educational practices and building some kind of relationship with their learners that motivates them and consequently increase their hope, as well. This description offers important applications for teachers, from a practical point of view. First, it demonstrates that perceived control is a fundamental precursor to emotions and language performance. When learners feel like they are in control of their education and appreciate accomplishment, positive feelings such as enjoyment of learning, hope, and pride are fostered, and negative feelings like fear, hopelessness, or boredom are reduced. Consequently, designers of FL intervention must take into consideration executing educational techniques and including intervention activities that increase students’ perception of control over their educational program to improve foreign language performance both directly and indirectly by improving the perception of control by nurturing and promoting positive feelings and decreasing negative ones (Hamm et al., 2017).

In addition, it is clear from the current review that English educators should take into account learners’ educational feelings when teaching English; if they do not do know how to deal with these feeling, they cannot reduce learners’ unpleasant feelings. So, these positive emotions can be investigated based on student-teacher interpersonal variables (Xie and Derakhshan, 2021). Educators must also understand and support the class’s psychological response to learning English and try to reduce their unpleasant feelings, such as frustration, fear, discouragement, and fatigue and they should feel free to express their feelings to their learners. Through empathetic learning, people can experience less fear, rage, discouragement, and more enjoyment and hope. Furthermore, learners’ self-regulation can be developed by educators by taking responsibility for them both in the school and in the educational setting. Hence, it is important to guide the learners to regulate their self-regulation both during classroom activities and in other settings. To make learning more fun, for instance, educators should change the way they interact with learners during the educational process. For instance, if learners feel uninterested, educators should change the way they learn to make the experience more enjoyable for them (Horton and Wallander, 2001).

Moreover, a significant cognitive-inspirational concept in the growth of positive psychology appears to be hope. The present review offered a preliminary suggestion that hope mirrors a psychological force that can cushion the influences of acute negative occurrences. hope seems to play a crucial role in the goal-setting cycle, as successful academic assignments demand a combination of inspiration and planning (Feldman and Kubota, 2015). Therefore, this review also provided further evidence of the significant functional task of inspirational cycles in maintaining learners’ enjoyment and hope under stressful living conditions. Also, learners who have a strong sense of hope establish attainable targets and make effective plans, relying on their abilities. Hope-filled learners are more expected to be able to regulate the academic environment and will have a more productive life in the long run. Language educators must create a setting through which learners feel safe and motivated to engage. Since pride in achievement also has a positive effect on the enjoyment of the english as a foreign language (EFL), teachers should assess students’ achievement and make them aware of their progress.

This review has also an implication for scholars as they can control the achievements of psychological researchers in positive emotions and positive psychology (PP) to broaden the research agenda regarding the function of positive emotion in language learning. To promote and adequately support continuous and rapid language learning, language experts and teachers must acknowledge the significance of feelings, and also take psychological factors into account. By helping learners set objectives and develop pathways and agency thinking, school psychologists play a unique role in enhancing learners’ hope. Additionally, psychology in schools can aid educators in permeating hope to the subject matter they are teaching, as well as work with parents to help the hope transmission process. Furthermore, more studies with different research designs such as mixed-methods can be carried out to contain data associated with hope, pride, and self-regulation, together to provide an extended view of the issue and lessen any inclinations. Implementing different types of interventions or treatments are recommended in further studies in illuminating positive emotions of learners in language learning.

## Author Contributions

XY has made a direct and intellectual contribution to the work, and got it ready for its publication.

## Conflict of Interest

The author declares that the research was conducted in the absence of any commercial or financial relationships that could be construed as a potential conflict of interest.

## Publisher’s Note

All claims expressed in this article are solely those of the authors and do not necessarily represent those of their affiliated organizations, or those of the publisher, the editors and the reviewers. Any product that may be evaluated in this article, or claim that may be made by its manufacturer, is not guaranteed or endorsed by the publisher.
